# Peer-Review of “Real-Time Health Monitoring Using 5G Networks: Deep Learning–Based Architecture for Remote Patient Care”

**DOI:** 10.2196/83423

**Published:** 2025-10-01

**Authors:** Shruti Bharadwaj

**Affiliations:** 1 United College of Engineering & Research, United Tower 53, Leader Road, Prayagraj, India, 91 1800 3131 808

**Keywords:** 5G, real-time patient monitoring, vital signs, prediction, deep learning, machine learning

*This is a peer-review report for “Real-Time Health Monitoring Using 5G Networks: Deep Learning–Based Architecture for Remote Patient Care.”*

## Round 1 Review

### Review Report

This paper [1] presents a novel architecture integrating deep learning and 5G networks to enhance real-time remote patient monitoring.

1. The combination of a convolutional neural network/long short-term memory model with 5G ultra-reliable low latency communication enables real-time monitoring with high accuracy and low latency. Achieving 96.5% accuracy for vital sign prediction demonstrates the effectiveness of the proposed model.
2. While tested on 1000 patients, analysis of its scalability to larger populations with diverse demographics would improve generalizability.
3. The use of attention mechanisms in the long short-term memory component improves the system’s ability to model dependencies in continuous vital sign monitoring.
4. A more detailed comparison with state-of-the-art remote monitoring systems, including their architectures and limitations, would strengthen the claims.
5. Since patient data is transmitted over 5G networks, an evaluation of encryption techniques, data integrity measures, and compliance with health care regulations (eg, the Health Insurance Portability and Accountability Act and the General Data Protection Regulation) should be included. Investigating performance under network congestion, packet loss, or fluctuations in 5G coverage would ensure system reliability.

### Final Recommendation

Accept with minor revisions.

This paper presents a promising and well-structured approach to real-time patient monitoring using deep learning and 5G technology. However, addressing concerns regarding computational efficiency, scalability, security, robustness, and explainability would further strengthen its impact.

### Suggested Revisions

- Include a comparative analysis with other remote patient monitoring systems.
- Provide details on computational resource use and energy efficiency for edge deployment.
- Address security, encryption, and data privacy considerations.
- Test and discuss model performance under varying network conditions.
